# Carnivores, competition and genetic connectivity in the Anthropocene

**DOI:** 10.1038/s41598-019-52904-0

**Published:** 2019-11-08

**Authors:** Scott Creel, Göran Spong, Matthew Becker, Chuma Simukonda, Anita Norman, Bastian Schiffthaler, Clive Chifunte

**Affiliations:** 1 Department of Ecology, 310 Lewis Hall, Montana State University, Bozeman, Montana, 59717 USA; 2 Institutionen för Vilt, Fisk och Miljö, Sveriges Lantbruksuniversitet, Skogsmarksgränd, 907 36 Umeå, Sweden; 3 Zambian Carnivore Programme, P.O.Box 90 Mfuwe, Eastern Province, Zambia; 4 Fisheries, Wildlife and Conservation Biology Program, Department of Forestry and Environmental Resources, North Carolina State University, 110 Brooks Ave, Raleigh, NC 27607 USA; 5 Zambia Department of National Parks and Wildlife, Private Bag 1, Kafue Road, Chilanga, Zambia; 6 Artedigränd 7, Fysiologisk botanik, UPSC, Umeå universitet, 901 87 Umeå, Sweden

**Keywords:** Ecology, Evolution

## Abstract

Current extinction rates are comparable to five prior mass extinctions in the earth’s history, and are strongly affected by human activities that have modified more than half of the earth’s terrestrial surface. Increasing human activity restricts animal movements and isolates formerly connected populations, a particular concern for the conservation of large carnivores, but no prior research has used high throughput sequencing in a standardized manner to examine genetic connectivity for multiple species of large carnivores and multiple ecosystems. Here, we used RAD SNP genotypes to test for differences in connectivity between multiple ecosystems for African wild dogs (*Lycaon pictus*) and lions (*Panthera leo*), and to test correlations between genetic distance, geographic distance and landscape resistance due to human activity. We found weaker connectivity, a stronger correlation between genetic distance and geographic distance, and a stronger correlation between genetic distance and landscape resistance for lions than for wild dogs, and propose a new hypothesis that adaptations to interspecific competition may help to explain differences in vulnerability to isolation by humans.

## Introduction

Current extinction rates are unprecedented in human history, and are comparable to five prior mass extinctions in the earth’s history^1^. Large mammals are among the most affected taxa^2^, and large carnivores have experienced particularly large declines in numbers and geographic distribution^3,4^. While many African protected areas have retained all of their large carnivores, the lion (*Panthera leo*), cheetah (*Acinonyx jubatus*) and African wild dog (*Lycaon pictus*) are all considered vulnerable or endangered^5–7^, and understanding the processes that allow demographic and genetic connections between small and increasingly isolated populations of such species is critical for their long-term conservation^8^.

Connections between ecosystems depend on their area and isolation^9,10^, which is increasingly determined by human activity^11,12^. More than half of the earth’s terrestrial surface has been modified by humans^13^, and extensive data from GPS tracking show that animal movements decrease as the human footprint increases^14^. Because of the concerns described above, large carnivores are well-represented in research on the ways that the human activities affect animal movement^15^ and connectivity between carnivore populations has been a focal point for many large-scale conservation efforts (*e.g*., the five-nation, 520,000 km^2^ Kavango-Zambezi Transfrontier Conservation Area)^16,17^. Despite these efforts, we are aware of no prior research that has applied standardized methods to test the relationship between landscape resistance and genetic connectivity for multiple large carnivores and multiple ecosystems. Connectivity between a given pair of ecosystems is affected not only by the landscape, but also by species’ traits^8^, and within the African large carnivore guild, well-documented responses to interspecific competition are likely to affect connectivity through opposing effects on individual movement and population density. In direct interactions between species, lions and spotted hyenas (*Crocuta crocuta*) are behaviorally dominant, while cheetahs and African wild dogs are subordinate, and populations of the dominant competitors invariably outnumber those of the subordinates^18–20^. Wild dogs and cheetahs reduce competition through diet partitioning, temporal segregation and spatial segregation^18,21–24^, and there is consistent evidence that they often persist within an ecosystem by avoiding unfavorable locations where competition is strong^18–20,24–26^.

**Figure 1:**
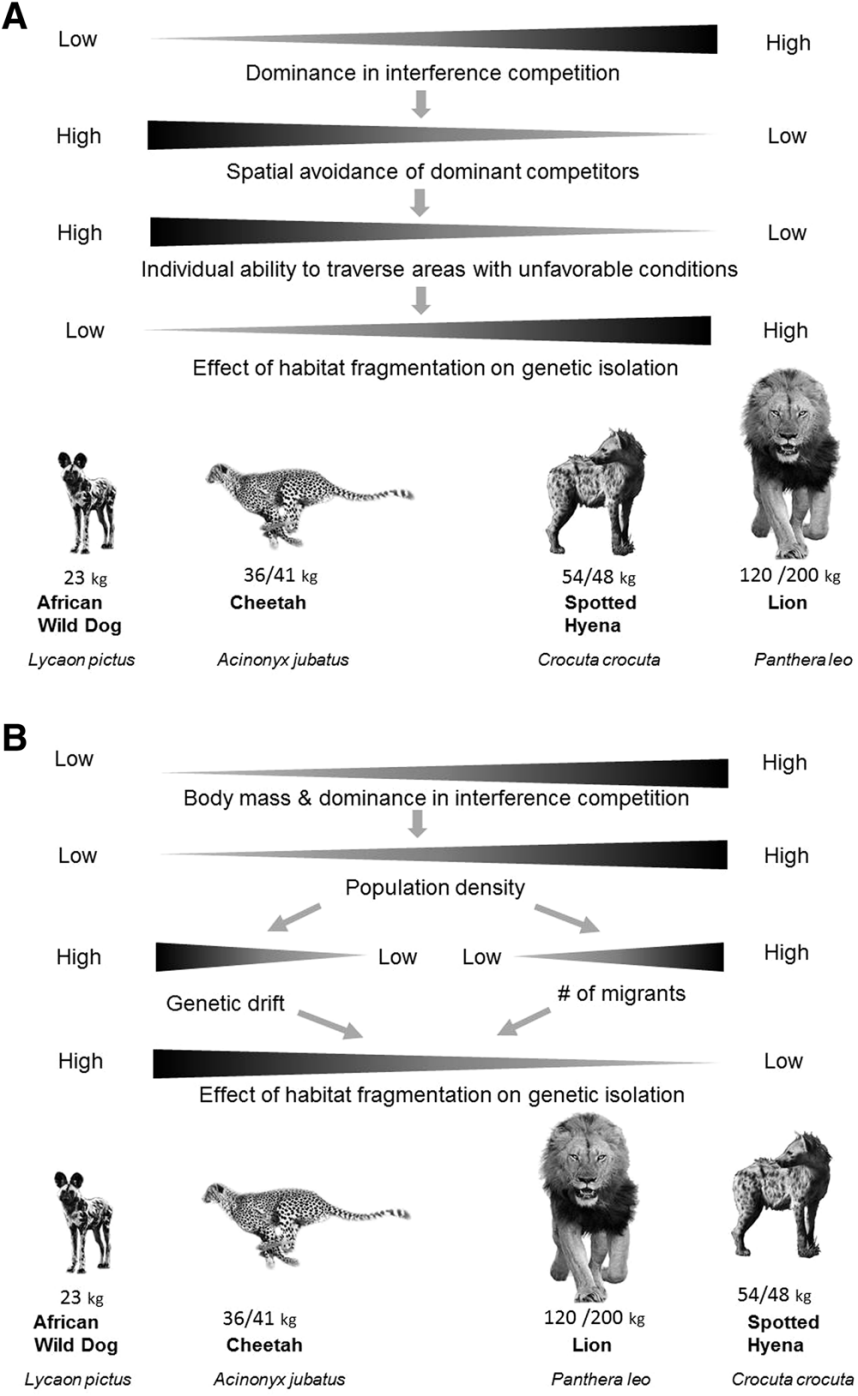
(**A**) The Competition-Movement-Connection Hypothesis predicts that, because adaptations of subordinate competitors allow them to traverse areas of the landscape with unfavorable ecological conditions, they will also be less isolated by anthropogenic habitat fragmentation. Thus we should detect (i) weaker correlations between anthropogenic landscape resistance and genetic distance, and (ii) weaker gradients of genetic distance between ecosystems for subordinate competitors, relative to dominant competitors. (**B**) In contrast, the Competition-Density-Connection Hypothesis predicts that, because dominant competitors attain higher population densities than subordinates (and because larger populations increase the number of potential dispersers and reduce genetic drift), we should detect (i) weaker correlations between anthropogenic landscape resistance and genetic distance and (ii) weaker gradients of genetic distance between ecosystems for dominant competitors, relative to subordinate competitors.

Little direct attention has been paid to the consequences of these effects for connectivity between ecosystems, but logic suggests that competition might affect connectivity through opposing effects on individual movement and population density (Fig. 1). The Competition-Movement-Connection hypothesis (Fig. 1A) suggests that the adaptations allowing subordinate competitors to move through areas with unfavorable competitive conditions might also allow them to move through areas made unfavorable by humans, and predicts that (i) landscape-scale genetic differentiation will be weaker for subordinate competitors, and (ii) correlations between anthropogenic landscape resistance and genetic differentiation will be weaker for subordinate competitors. Broadly, the Competition-Movement-Connection hypothesis focuses on the effect of species’ traits on connectivity^27^. More narrowly, it identifies interspecific competition as a strong limiting effect for subordinate competitors in this guild, and suggests that the adaptations that allow subordinates to avoid unfavorable locations within ecosystems^28^ will also facilitate connectivity between ecosystems, if the adaptations are sufficiently general (for example, if the speed or linearity of movements increases when unfavorable conditions are encountered). In contrast, the Competition-Density-Connection hypothesis notes that dominant competitors within this guild attain higher population densities, and (because larger populations provide more potential dispersers and experience less genetic drift) predicts that (i) landscape-scale genetic differentiation will be weaker for dominant competitors, and (ii) correlations between anthropogenic landscape resistance and genetic differentiation will be weaker for dominant competitors (Fig. 1B). Although the evolutionary processes and traits that most strongly affect connectivity are likely to vary across taxa, interspecific competition is ubiquitous^29^ and often leads to spatial niche partitioning^30,31^, so these hypotheses may have broad applicability.

## Brief Methods

We tested these predictions using SNP genotypes for African wild dogs (96 individuals, 2,584 loci, 3 ecosystems) and lions (208 individuals, 3,528 loci, 3 ecosystems) in Zambia (Liuwa, Kafue and South Luangwa) and southern Tanzania (Selous). Briefly (see full methods below), we collected tissue samples and recorded locations (shown in Fig. 3) by GPS. We extracted DNA and used high throughput sequencing to identify and score restriction-site associated single nucleotide polymorphisms (SNPs). Genome-wide SNP genotypes are sensitive to current gene flow (Fig. S1), and thus avoid the possibility of mistaking genetic similarities that arose prior to human effects as evidence that the landscape still provides connectivity. Using R, we determined genetic distance using the SNPRelate package^32^, and quantified spatial variation in SNP genotypes using t-distributed stochastic neighbor embedding^33,34^ and spatial principal components analysis (sPCA)^35–37^. We quantified anthropogenic landscape resistance between ecosystems by fitting a circuit model to the Human Footprint Index (HFI), which provides ground-truthed one km^2^ resolution mapping of built environments, crop lands, pasture lands, human population density, night lights, railways, roadways, and navigable waterways^11^. The HFI combines these effects in a single measure of human alteration of the landscape that has been shown to detect effects on animal movement^14^ and allows repeatable analysis for other species and ecosystems. Prior validation of the HFI confirms that it has low root-mean-squared-error for the biomes in this study^11,38^. Finally, we tested correlations between genetic distance, geographic distance and landscape resistance using Mantel tests^39^.

## Results

**Figure 2:**
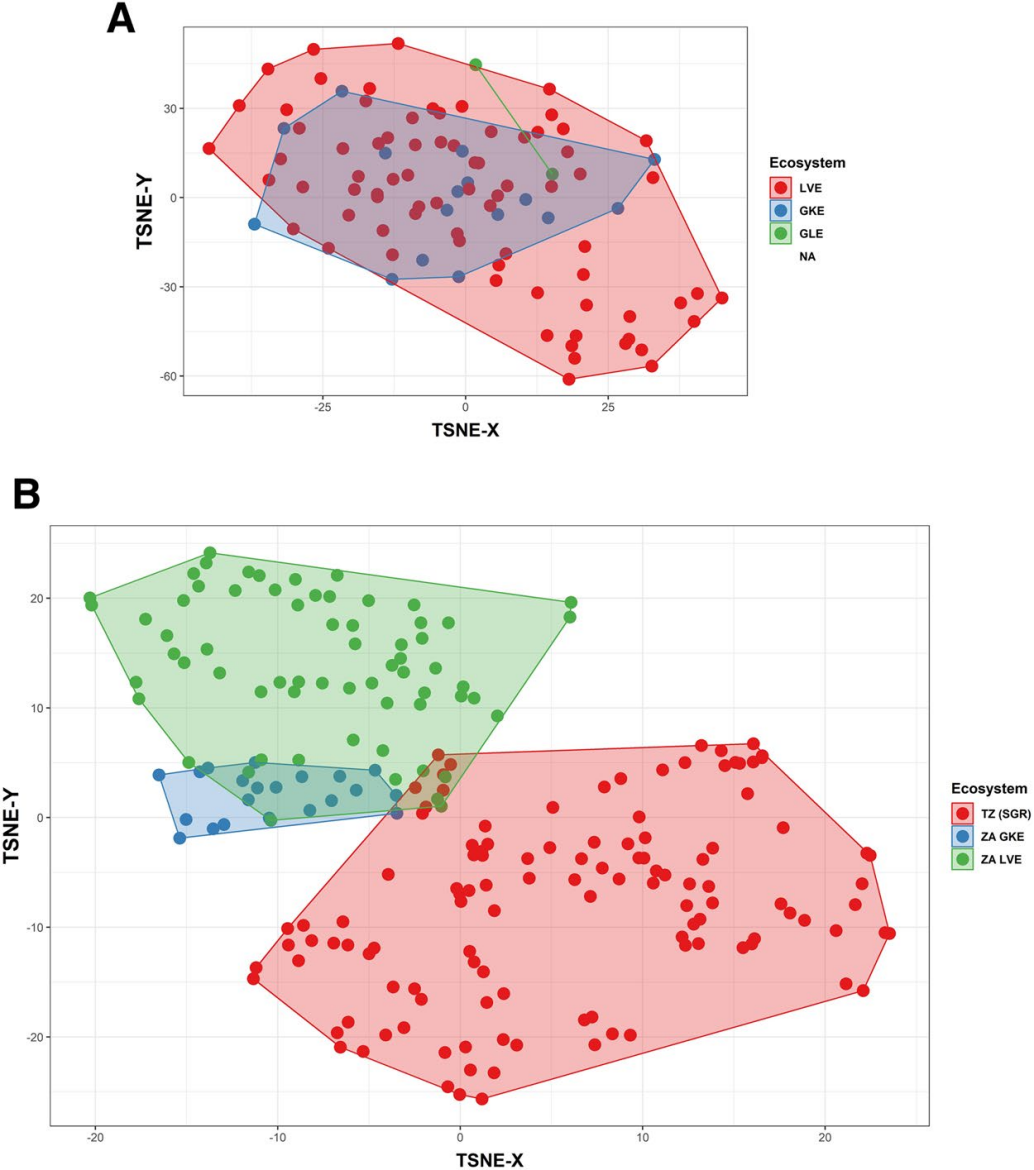
Ordination of SNP genotypes by t-distributed stochastic neighbor embedding, for (**A**) African wild dogs and (**B**) lions. Colors show convex hulls connecting genotypes from a given ecosystem. LVE = Luangwa Valley Ecosystem, GKE = Greater Kafue Ecosystem, GLE = Greater Liuwa Ecosystem, SGR = Selous Game Reserve.

**Figure 3:**
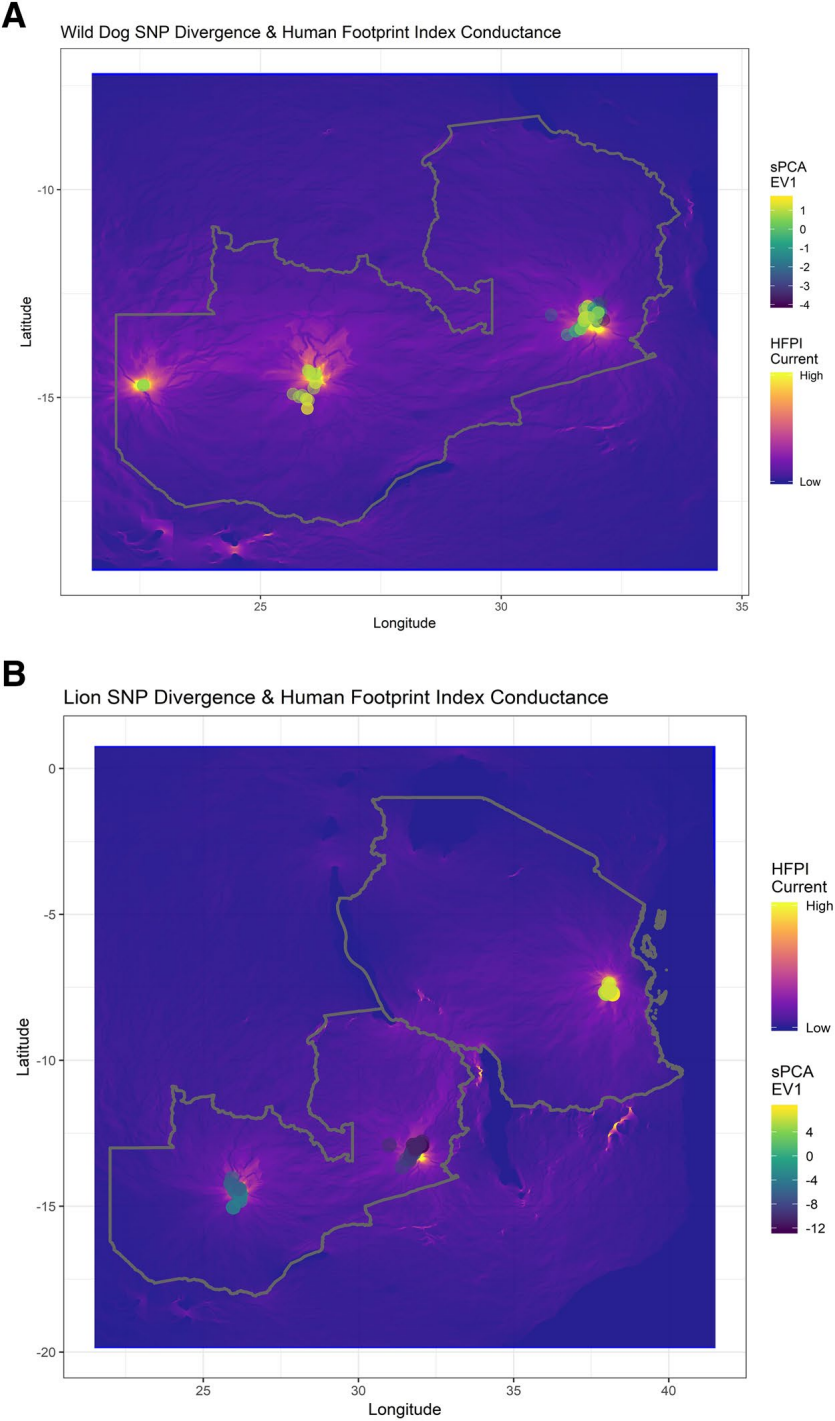
Patterns of genetic variation from spatial principal components analysis superimposed on landscape resistance from a circuit model fit to the Human Footprint Index. Differences in point color reflect differences in the first eigenvector of sPCA fit to SNP genotypes for (**A**) wild dogs and (**B**) lions. In the underlying circuit model, cold colors map areas with low current (high anthropogenic resistance) and warm colors map areas with high current (low anthropogenic resistance).

African wild dogs showed considerably stronger connectivity between ecosystems than lions, suggesting that a strong capacity for individual movement overrides the effects of low population density. Ordination of SNP genotypes with t-distributed stochastic neighbor embedding revealed extensive genetic overlap between ecosystems for wild dogs and little genetic overlap for lions (Fig. 2). Spatial PCA also revealed larger (3.6-fold) genetic differences between ecosystems for lions than for wild dogs (Fig. 3, inter-ecosystem range of sPCA eigenvector one scores: lions = 20.33, wild dogs = 5.63). The correlation between genetic distance and geographic distance was weak for wild dogs (Mantel = −0.04, *P* = 0.78) but strong for lions (Mantel = 0.39, *P* < 0.001). The correlation between genetic distance and anthropogenic landscape resistance (Fig. 3) was also weak for wild dogs (Mantel = −0.05, *P* = 0.85) but strong for lions (Mantel = 0.55, *P* < 0.001).

## Discussion

Interspecific competition affects virtually all species^40–43^ and has long been recognized as an important force structuring large carnivore guilds^44–46^. Our results suggest that the adaptations of subordinate competitors may increase connectivity between ecosystems by promoting their ability to move through areas with unfavorable conditions^19,24,47^, even though competition holds them at low population densities^18,20,25^.

Our analyses compared three ecosystems for each species and measured geographic distance and anthropogenic resistance between each pair of ecosystems, but only two ecosystems (Luangwa and Kafue) were sampled for both species. We did not examine lions in Liuwa because this population is mainly derived from lions translocated from Kafue. Southern Tanzania’s Selous Game Reserve provided an alternative lion population with similar isolation by distance and anthropogenic resistance, and all of the patterns hold if one restricts attention only to Kafue and Luangwa (Figs 2 and 3). Translocation can be used as a conservation tool to improve connectivity, but it supplants natural processes of gene flow and obscures the genetic patterns that result (and with the emergence of illegal trafficking in big cat body parts in Africa^48^, translocations complicate the task of identifying the sources of traffic). Fencing can also be used as a conservation tool for small populations^49,50^, with obvious consequences for connectivity. In this study, we examined only populations that have not been affected by fencing or translocation.

Legal and illegal human hunting can drive local source-sink dynamics in lions and other carnivores, both by drawing individuals out of protected areas to vacant territories where hunting has occurred (promoting movement), by increasing mortality rates just outside protected areas (impeding movement), or by decreasing population density (also decreasing connectivity)^51–55^. Because variation in the distribution and intensity of human hunting is not captured by changes in the physical landscape, its potential effects on connectivity were not included in this analysis. Further study is warranted to test whether its effects within ecosystems propagate into connectivity between ecosystems.

RAD SNP genotypes at thousands of loci contain enough information to detect recent restrictions in gene flow (Supplemental Fig. S1), but they include genetic differences of both ancient and recent origin. Given this, it is revealing that for lions, genetic distance was more strongly correlated with anthropogenic resistance (Mantel correlation = 0.55) than with geographic distance (Mantel correlation = 0.39). This pattern strongly suggests that human activity has already affected genome-wide genetic differentiation between lion populations. The lack of correlation for wild dogs in a parallel test suggests that wild dogs have maintained more natural large-scale patterns of movement, and confirms the importance of multi-species tests. The strong correlation between genetic distance and anthropogenic landscape resistance for lions could have been detected by a single-species study, but multi-species studies are needed to understand how species’ traits affect connectivity.

While conclusive tests of the Competition-Movement-Connection and Competition-Density-Connection hypotheses will require data from more species and ecosystems, our analyses show that efforts to maintain connectivity for small, isolated populations cannot rely on the assumption that one approach will work equally well for all species, even for pairs of species with substantial overlap in their ecology. As in our study, stronghold populations of several endangered carnivores overlap for other guilds (e.g. the wolf, lynx, wolverine and brown bear in North America and Scandinavia, and the tiger, leopard and dhole in Asia) and the hypothesis that subordinate competitors are better connected than dominant competitors (despite smaller populations) warrants testing with other species and locations. More broadly, it is well established that species’ traits affect connectivity, but there is considerable scope for better understanding of the traits and processes that most affect connectivity. Finally, our analysis shows that the human footprint affects not only short-term movements^14^, but also the patterns of genetic differentiation that arise if effects on movement are consistent over time. For lions, landscape resistance due to the human footprint provides a better explanation of genetic differentiation than geographic distance does, revealing that their evolution has already been modified by Anthropocene processes.

## Full Methods

### Sampling and extraction of DNA

As part of long-term field studies conducted with methods approved by the Zambia Department of National Parks and Wildlife and the Tanzania Commission for Science and Technology and Department of Wildlife, we collected tissue biopsies in the field when individuals were anaesthetized for radiocollaring or snare removal. Other individuals were sampled with little disturbance using biopsy darts (PneuDart) fired from a stationary vehicle with a Dan-Inject JM Standard air rifle. Within Zambia, samples were collected only by licensed wildlife veterinarians and within Tanzania samples were collected only by trained personnel with extensive experience using biopsy darts, following guidelines of the Department of National Parks and Wildlife (Zambia), and the Commission for Science and Technology (Tanzania) approved by the Montana State University Animal Care and Use Committee (protocol 2010-39). Samples were immediately transferred to ethanol for storage. We extracted DNA at our Luangwa field site using the QIAsymphony DNA kit (Qiagen) according to manufacturer’s instructions. Extract quantity and purity were assessed at our lab in Sweden initially using a spectrophotometer (NanoDrop, Thermo Fisher Scientific). DNA quality for sequencing was further screened by gel electrophoresis using the Kodak Electrophoresis Documentation and Analysis System 120 (Eastman Kodak), and finally screened using a Qubit prior to library preparation and sequencing at the National Genomics Institute, Stockholm.

### Library preparation, sequencing and scoring SNP genotypes

DNA extracts from lions and wild dogs were sequenced using DNA associated with the restriction site for EcoR1, optimizing the trade-off between breadth of coverage (≈1% of the genome) and read depth (minimum of 30 × /nucleotide). Specifically, we used EcoR1 to digest 0.5 μg of each DNA extract according to manufacturer’s instructions. We removed the activated enzyme using the MinElute Reaction Cleanup kit (Qiagen) in two elutions, and visualized the second elution by gel electrophoresis to assess the quality of the digestion. Satisfactory samples (91%) were sent to the National Genomics Institute (Stockholm) for library construction and preparation. Fragments 400 to 700 bp were excised and blunt end repaired. Paired-end, multiplexed adapters were ligated to the fragments by sample, and equimolar concentrations were sequenced on an Illumina HiSeq. 2500 or NovaSeq, resulting in 2 × 150 bp paired-end reads. We demultiplexed sequenced Illumina reads using the barcode_splitter option of the FASTX Toolkit (v 0.0.13) and removed adapters with cutadapt (v 0.9.3; [36]). Reads were trimmed and quality filtered using the FASTX Toolkit trimmer and quality_filter options respectively using the settings: q 10, p 70. Sequence quality was assessed using FastQC (v 0.9; Babraham Bioinformatics). Sequences were used as input for SNP detection using Stacks (v. 2.0), with settings m 5–10, M 4, n 3. These procedures yielded genotypes for 3,528 SNP loci from 205 lions in 3 ecosystems, and for 2,584 loci from 96 wild dogs in 3 ecosystems.

### Statistical methods

SNP genotypes from the population_haplotypes output of Stacks were recoded to snpgds format in R following procedures described by Zheng at http://corearray.sourceforge.net/tutorials/SNPRelate/. We examined spatial variation in genotypes using ordination by t-distributed stochastic neighbor embedding. We confirmed that patterns of similarity and difference between ecosystems from t-SNE were stable across iterations and were the same as those produced by the PCA procedures of the SNPRelate package. We also used spatial PCA to describe spatial variation in genotypes. We identified the sPCA components to retain for each species following procedures described by Jombart at https://github.com/thibautjombart/adegenet/wiki/Tutorials, retaining one global component in the sPCA for lions (Supplemental Fig. S2) and two global and one local components in the sPCA for wild dogs.

We calculated genetic distance across all loci as the Euclidean distance from the first three t-SNE components, and compared these to matrices of geographic distance and anthropogenic landscape resistance using Mantel tests. We obtained anthropogenic landscape resistance values by downloading the global Human Footprint Index dataset from https://datadryad.org/resource/doi:10.5061/dryad.052q5, subsetting a raster for Zambia (wild dogs) or Zambia and Tanzania (lions), and using CircuitScape to obtain current maps linking each pair of ecosystems (with zero resistance within ecosystems). Pairwise resistance values from CircuitScape were used as entries in the landscape resistance matrix.

## Supplementary information

Dataset 1

Dataset 2

Dataset 3

Dataset 4

Dataset 5

Dataset 6

Supplementary Tables and Figures

## Data Availability

The sequence data are subject to a memorandum of understanding with the Zambia Department of National Parks and Wildlife, to whom queries should be directed. Matrices with pairwise genetic distance, geographic distance and landscape resistance are included in the Supplementary Materials.

## Code availabilty

R code used in analysis is available by request to screel@montana.edu.
