# Supplementary material for: Carnivores, competition and genetic connectivity in the Anthropocene
Source: Sci Rep. 2019 Nov 8;9:16339. doi: 10.1038/s41598-019-52904-0 (PMC6841969; doi:10.1038/s41598-019-52904-0)
Supplement: Supplementary file 7 — Supplementary Tables and Figures [file 41598_2019_52904_MOESM7_ESM.pdf]

Supplementary Information for

Carnivores, competition and genetic connectivity in the Anthropocene

Scott Creel[1,2,3,*], Göran Spong[2,3,4], Matthew Becker[3], Chuma Simukonda[5], Anita Norman[2], Bastian Schiffthaler[6], Clive Chifunte[5]

1 Department of Ecology, 310 Lewis Hall, Montana State University, Bozeman, Montana 59717 U.S.A.

2 Institutionen för Vilt, Fisk och Miljö, Sveriges Lantbruksuniversitet, Skogsmarksgränd, 907 36 Umeå, Sweden

3 Zambian Carnivore Programme, P.O.Box 90 Mfuwe, Eastern Province, Zambia

4 Fisheries, Wildlife and Conservation Biology Program, Department of Forestry and Environmental Resources, North Carolina State University, 110 Brooks Ave, Raleigh, NC 27607, U.S.A.

5 Zambia Department of National Parks and Wildlife, Private Bag 1, Kafue Road, Chilanga, Zambia.

6 Artedigränd 7, Fysiologisk botanik, UPSC, Umeå universitet, 901 87 Umeå

* Correspondence to screel@montana.edu

Supplementary Material

Matrices with genetic distance for all pairwise combinations of individuals are saved as the supplemental files genmat_lion.csv and genmat_wd.csv. Matrices with pairwise geographic distance are in the supplemental file geomat_lion.csv and geomat_wd.csv. Matrices with pairwise anthropogenic resistance are in the supplemental files resmat_lion.csv and resmat_wd.csv.

Supplementary Figures

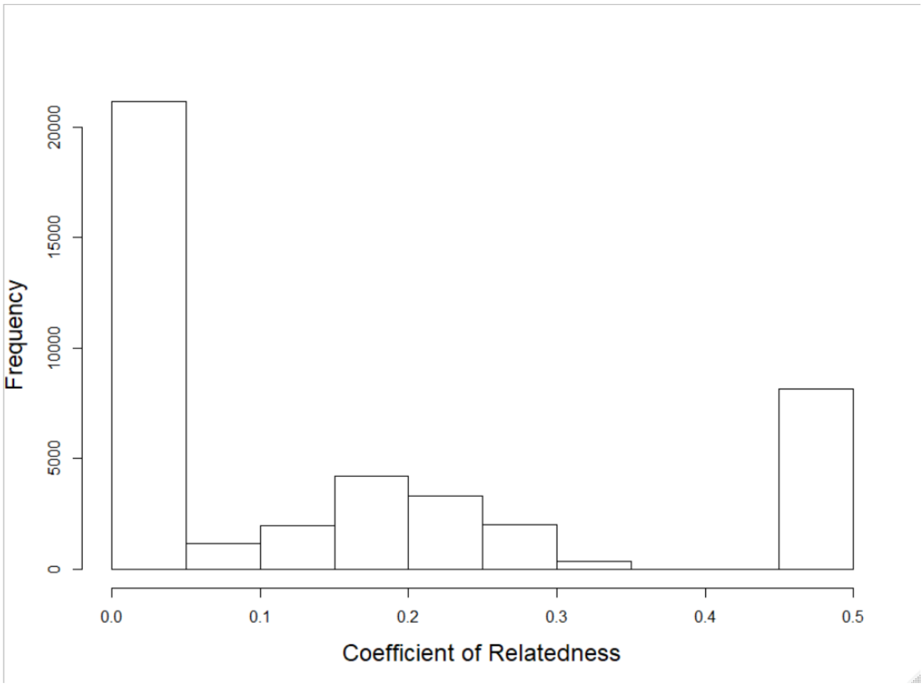

Fig. S1. SNP genotypes from restriction site associated DNA provide information to detect recent effects on genetic patterns, as demonstrated by their power to distinguish pairs of first-order (r = 0.5) relatives from second-order (r = 0.25) relatives and unrelated individuals, shown here with the frequency distribution of the coefficient of relatedness between all pairs of lions. The pairwise matrix of identity-by-descent that underlies these coefficients of relatedness is highly similar to the pairwise matrix of identity-by-state for these data, empirically confirming that these markers evolve rapidly enough to show the signal of recent effects on gene flow.

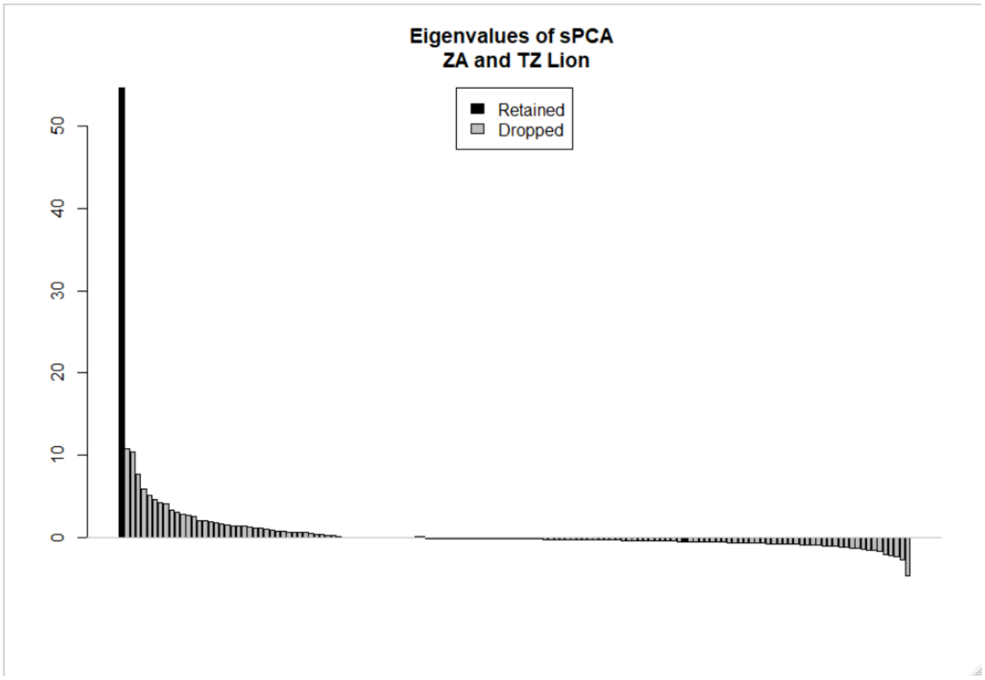

Fig. S2.

The distribution of eigenvalues for positive and negative sPCA components provided clear guidance for components to be included in sPCA scores following the method recommended by Jombart. In the example shown for lions, only the first positive component, which was more than five times larger than all other components, was retained.
